# Detection of Taiqiu sweet persimmons during the color-transition period with an improved YOLO11-FC2T model and causal analysis

**DOI:** 10.3389/fpls.2025.1742794

**Published:** 2026-01-27

**Authors:** Wenhui Dong, Huiqin Li, Lifei Gao, Pengzhi Hou, Yaqing Zhi, Xiaoying Zhang

**Affiliations:** Faculty of Software Technologies, Shanxi Agricultural University, Taigu, China

**Keywords:** ATE, deep learning, DiffuseMix, tail-end dataset, YOLO11

## Abstract

**Introduction:**

Accurate detection of Taiqiu sweet persimmon in orchards is essential for estimating yield, planning harvest operations, and supporting intelligent management in precision agriculture. However, current fruit-detection approaches for this cultivar, especially during the color-transition period, suffer from highly subjective and inefficient manual inspection and from poor adaptability of existing deep-learning models to complex field scenes.

**Methods:**

In this study, we propose an improved YOLO11-based detector, YOLO11-FC2T, for robust detection under conditions with strong color–background coupling, small or adherent fruits, and uneven illumination. YOLO11-FC2T introduces four key architectural modifications: (1) a C3k2_FasterBlock to improve gradient-efficient feature learning; (2) a C2PSA_CGA module to enhance channel–spatial focus via coordinate-guided aggregation; (3) a three-layer Dysample-T structure to strengthen multi-scale representation; and (4) a cross-scale attention fusion module, CAFMAttention, to better decouple fruits from cluttered backgrounds. To further enhance generalization in complex orchard scenes without additional labeling cost, we introduced the DiffuseMix data-augmentation method and apply it to color-transition images.

**Results:**

Experiments show that YOLO11-FC2T clearly outperforms the YOLO11 baseline. The model achieves a precision of 91.7% (+1.0%), recall of 86.7% (+2.8%), mAP@0.5 of 94.8% (+1.6%), and mAP@0.5-0.95 of 81.2% (+4.0%), where mAP@0.5 uses an IoU threshold of 0.50. On a challenging tail-case set of 537 images, the false detection rate is 1.30%, with a 45.2% reduction in errors relative to YOLO11. In the performance evaluation stage, we first perform causal-effect analysis based on the Average Treatment Effect (ATE) to quantify the independent and joint contributions of each architectural component and of DiffuseMix; at the same time, the efficiency of the model is analyzed by the number of parameters (Params, M) and per-image inference latency (ms). in addition, we construct and use a dedicated tail-case dataset as a supplementary experiment to further verify the robustness and effectiveness of these improvements in the most difficult scenes. Finally, we introduced cross-condition test set to further validate the generalization capability of YOLO11-FC2T. The above results indicate that YOLO11-FC2T not only improves the indicators, but also possesses reliable generalization ability and stability.

**Discussion:**

Overall, YOLO11-FC2T addresses key detection challenges during the color-transition period and provides a practical, portable solution for automated fruit identification and counting in precision agriculture. The above results indicate that YOLO11-FC2T not only improves the indicators, but also possesses reliable generalization ability and stability.

## 1 Introduction

Taiqiu sweet persimmons are noted for their crisp texture and sweet flavor.The ripening process is highly sensitive to environmental conditions, especially during the color-transition period—the optimal harvest window and a key indicator for forecasting market supply (Cui et al., 2024). However, manual identification at this stage is subjective, inefficient for large-scale orchard monitoring, and sensitive to environmental variation, often leading to yield misestimation. These limitations undermine the profitability of the persimmon industry. Therefore, automated detection capable of identifying and counting Taiqiu sweet persimmons during the color-transition is urgently needed to enable stable, efficient and accurate fruit detection.

Detecting Taiqiu sweet persimmons during the color-transition period presents distinct technical challenges. First, the minimal chromatic difference between fruit (light yellow–green) and foliage (light to dark green) induces strong color–background coupling and very low global contrast (Jayita et al., 2023; Ziang et al., 2023), making it highly likely for the model to misidentify leaves as fruits. Second, the coexistence of small, distant fruit and tightly clustered fruit frequently causes both missed detections and false positives (Liu et al., 2024). Third, uneven illumination and stochastic occlusion from branches and leaves further complicate reliable recognition in orchards. Although, existing studies have made progress in areas such as model lightweighting, multimodal fusion, and attention mechanisms (Liu et al., 2018; Yang et al., 2023; Song et al., 2025), a systematic solution tailored to this growth stage remains underdeveloped. Many studies prioritize accuracy while constraining annotation cost yet still fail to improve generalization under complex lighting and low-contrast conditions; they also lack quantitative analysis of each module’s independent contribution and interaction effects, often reporting aggregate metrics only and providing limited explanatory evidence for why the methods are effective. This gap is central to effective detection during the transition period.

To address this gap, we propose YOLO11-FC2T, an improved YOLO11-based detector for Taiqiu sweet persimmons during the color-transition. The main enhancements are:

1. introducing FasterBlock into the backbone’s C3k2 to form C3k2_FasterBlock, improving feature-extraction efficiency and sensitivity to subtle fruit–leaf chromatic differences, reduce false detection caused by small color differences between fruits and leaves (Wang J. et al., 2024);
2. replacing the attention module in C2PSA with CGAAttention to construct C2PSA_CGA, enhancing focus on salient fruit regions and suppressing background interference, enabling the model to prioritize fruit targets over background elements (Wang J. et al., 2025).;
3. redesigning DySample as a three-layer structure (DySample-T) that adaptively adjusts sampling weights to better represent small and adherent fruit (Wang Q. et al., 2025);
4. incorporating a cross-scale attention fusion module (CAFMAttention) to promote multi-scale interaction and improve target separation in low-contrast scenes (Yao et al., 2025).

To reduce annotation cost, we introduced the DiffuseMix data-augmentation strategy, which yields more realistic and diverse samples than conventional augmentations (e.g., rotation and exposure adjustment) and markedly improves generalization under complex lighting and low-contrast conditions (Islam et al., 2024). Meanwhile, from a causal-inference perspective, we estimate the Average Treatment Effect (ATE) to quantify each module’s independent contribution and synergistic gains while controlling for training variability and inter-module interference (Cai et al., 2025). This causal approach complements precision, recall, and mAP by providing explanatory validation of effectiveness and a theoretical basis for practical orchard deployment. Finally, in addition to precision, recall, and mAP, we evaluated the number of parameters and per-image inference latency of YOLO11 and YOLO11-FC2T to assess the models’ resource consumption and response performance.

## 2 Related work

In agricultural automated detection, recent advances in deep-learning optimization and lightweight architectures have markedly improved crop and fruit recognition. Most studies focus on improving recognition accuracy and efficiency under task-specific settings. Yang et al. (2022) integrated the Tiny Hourglass-24 lightweight backbone into an anchor-free CenterNet and refined residual modules to improve speed and accuracy for multi-apple detection in dense orchards; nevertheless, performance declined in distant, dense scenes with smaller, overlapping targets (AP decreased from 98.90% to 93.63%), indicating limited robustness under severe occlusion. To address high weed density, low recognition rates, and potential yield impacts during the soybean seedling stage, Cui et al. (2024) developed a weed-identification method using low-altitude UAV imagery with Faster R-CNN. They curated a 4000-image PASCAL VOC–format dataset, adopted VGG16 as the backbone, and optimized anchor boxes. The model achieved 88.69% single-frame accuracy with a per-image inference time of 310 ms, demonstrating potential for UAV-based weed control. Despite these advances, scene adaptability remains limited: the former degrades in distant, dense, or occluded scenarios, whereas the latter lacks explicit validation under extreme environments and evidence for large-scale deployment. Thus, robustness under complex field conditions and natural interference requires further improvement.

To address limitations in adapting to complex agricultural scenes, the YOLO family—valued for its balance of speed and accuracy—has become a central focus for algorithmic improvements and scenario-specific adaptations. To mitigate low accuracy and missed detections caused by leaf occlusion and variable lighting in greenhouses, Zheng et al. (2024) developed YOLOv8-Tomato, an enhanced YOLOv8n-based model that integrates LSKA attention into the SPPF layer to strengthen local feature capture, replaces conventional interpolation with the lightweight dynamic upsampler DySample to improve feature reconstruction, and adopts the Inner-IoU loss to accelerate box regression, collectively improving accuracy while preserving efficiency. Hui et al. (2024) improved YOLOv7 for low-light conditions by introducing a weakly supervised adaptive scheme with adaptive data augmentation, thereby enhancing detection under dim lighting. For fruit-and-stem detection in complex orchard backgrounds, Den (Deng et al., 2024) integrated BiFormer attention into YOLOv7 and employed WIoU for box regression, enhancing small-target discrimination amid background noise while improving accuracy and real-time performance. Zhang et al. (2025) proposed an improved framework, YOLOv11-4ConvNeXtV2, based on YOLOv11 and ConvNeXtV2 to enhance persimmon ripeness detection accuracy. This framework integrates the ConvNeXtV2 backbone network, fully convolutional mask autoencoder (FCMAE) pre-training, global response normalization (GRN), and single-head self-attention (SHSA) mechanism, significantly improving the detection capability of small objects under ambiguous, occluded, and complex lighting conditions.To address the issue of color characteristics being easily affected by light interference and occlusion during tomato ripening, Li et al. (2023) proposed the MHSA-YOLOv8 model. By introducing a multi-head self-attention mechanism (MHSA) at the backbone network’s end, the model enhances its ability to extract and distinguish color differences in tomatoes at different ripeness stages through multi-dimensional grouped attention operations. Particularly for semi-ripe tomatoes and ripe tomatoes, which exhibit similar color characteristics due to light interference, the MHSA mechanism accurately captures subtle differences in color distribution between the two fruit types, thereby improving feature recognition accuracy.

However, these methods are largely tailored to conventional orchard settings and remain inadequate for the unique requirements of Taiqiu sweet persimmons during the color-transition period—a stage that poses greater technical challenges than standard scenarios—necessitating further targeted investigation.

## 3 Materials and methods

### 3.1 Data collection

**Figure 1:**
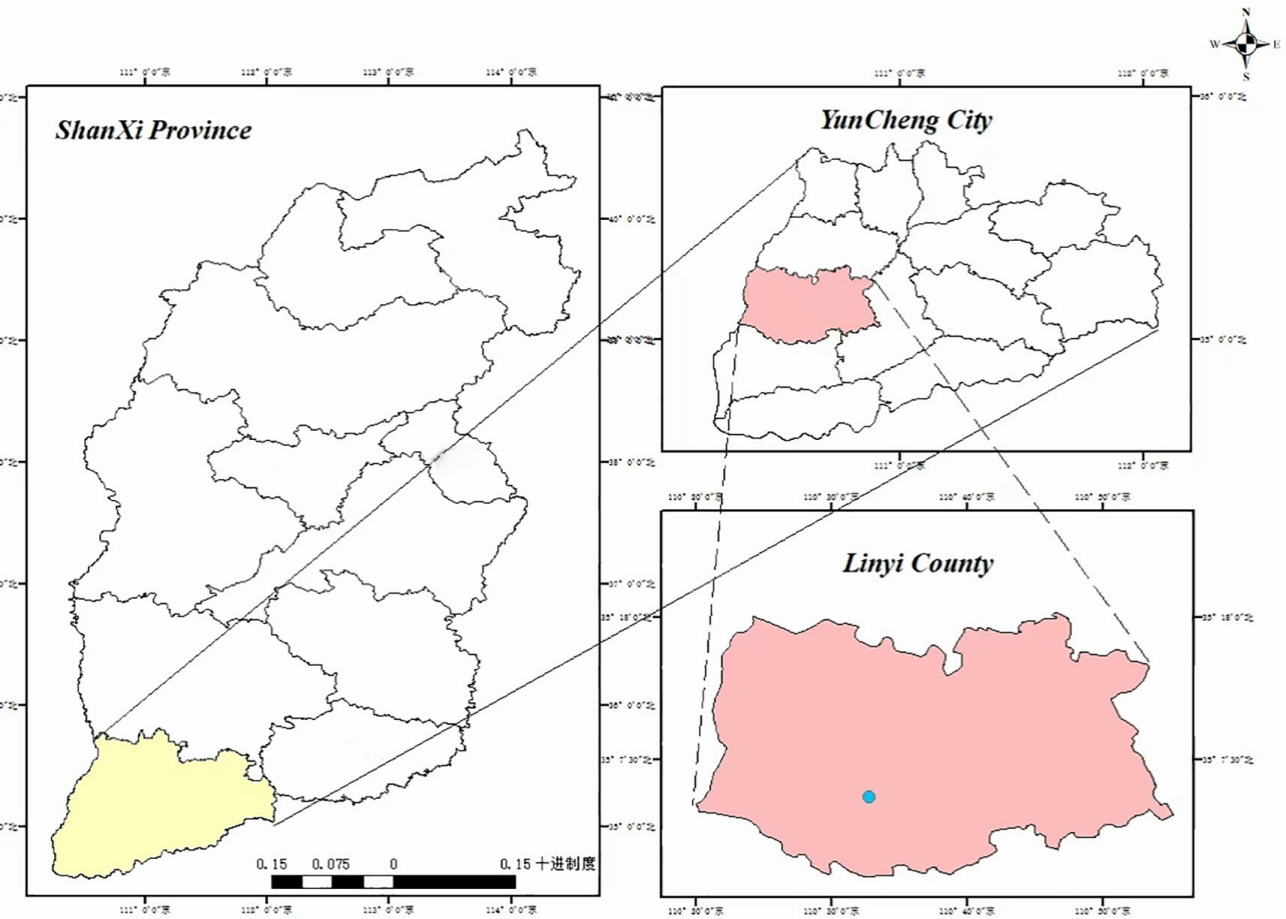
Data collection sites for Taiqiu sweet persimmons.

The primary dataset used in this study was collected from the Shen’ai fruit tree cultivation base in Linyi County, Yuncheng City, Shanxi Province, as shown in **Figure 1**. To ensure data consistency and controllability, a standardized image acquisition protocol was adopted: starting from the initial color-transition stage of the fruits, systematic collection was conducted at 10-day intervals, with strictly controlled shooting distances (30, 45, and 60 cm), multiple viewpoints, and typical illumination conditions including both front-light and back-light scenarios. All images in the primary dataset were captured using an iPhone 13 Pro Max. This standardized acquisition strategy effectively reduces potential interference caused by variations in distance, viewpoint, and acquisition timing, thereby ensuring a stable and controlled data foundation for model training, comparative experiments, and ablation studies.

To evaluate the model’s generalization ability across different acquisition regions and environmental conditions, we additionally constructed a cross-condition test set, which was collected from an orchard in Yuanjiazhuang Village, Linyi County, Yuncheng City, Shanxi Province, containing approximately 400 annotated images. In contrast to the primary dataset, the cross-condition test set exhibits notable differences in acquisition conditions:

1. Acquisition device — the cross-condition test set was captured using a Samsung Galaxy S24 Ultra, whereas the primary dataset was captured with an iPhone 13 Pro Max. Due to differences in camera optics (sensor size and lens characteristics), image signal processing pipelines (color reproduction, white balance strategies, sharpening), and exposure control mechanisms between devices, the resulting images exhibit noticeable domain shifts in tone distribution, texture detail, and noise patterns. Such discrepancies reflect realistic deployment scenarios where multiple mobile devices may be used, enabling a more rigorous evaluation of the model’s robustness and generalization ability under heterogeneous hardware conditions.
2. Acquisition mode — while the primary dataset was collected following a standardized protocol with fixed shooting distances, the cross-condition test set was obtained randomly during daily orchard patrols, with no strict constraints on shooting distance, viewpoints, or illumination, making it more reflective of real-world application scenarios.

It is important to note that the cross-condition test set did not participate in model training, validation, or hyperparameter tuning. It was used exclusively for external testing to avoid potential data leakage and to enable an objective evaluation of the model’s cross-region and cross-condition generalization ability.

### 3.2 Data processing

#### 3.2.1 Introduction to DiffuseMix

During the color-transition period, Taiqiu sweet persimmon fruit displays hues that closely approximate those of the foliage, and field lighting varies markedly—from intense illumination and backlighting to dappled backgrounds (Fang et al., 2025). Relying solely on conventional augmentations—rotation, exposure adjustment, translation, scaling, shearing, and mirroring—leads to two major issues: first, augmented samples can deviate from the true data distribution, causing the model to conflate “leaf texture” with “fruit cues”; second, blending-based augmentations (e.g., MixUp, CutMix) introduce label ambiguity (Gao et al., 2017). Moreover, diffusion models that synthesize entire images from scratch may lack photorealism or yield semantic label inconsistencies. To address these limitations, we introduce DiffuseMix (Islam et al., 2024), a data-augmentation strategy that increases data diversity and model robustness while preserving realism and label consistency.

DiffuseMix first employs a diffusion model to select k prompts from a predefined set. Using predefined prompts *p*_j_ (j∈[1,k]), it generates diverse variants *I*_ijvw_ that closely resemble the original image I_i_ in style, background, and details: As shown in Equation 1:

(1) $${\hat{I}}_{ijvw}=G(I_{i},p_{j})$$

**Figure 2:**
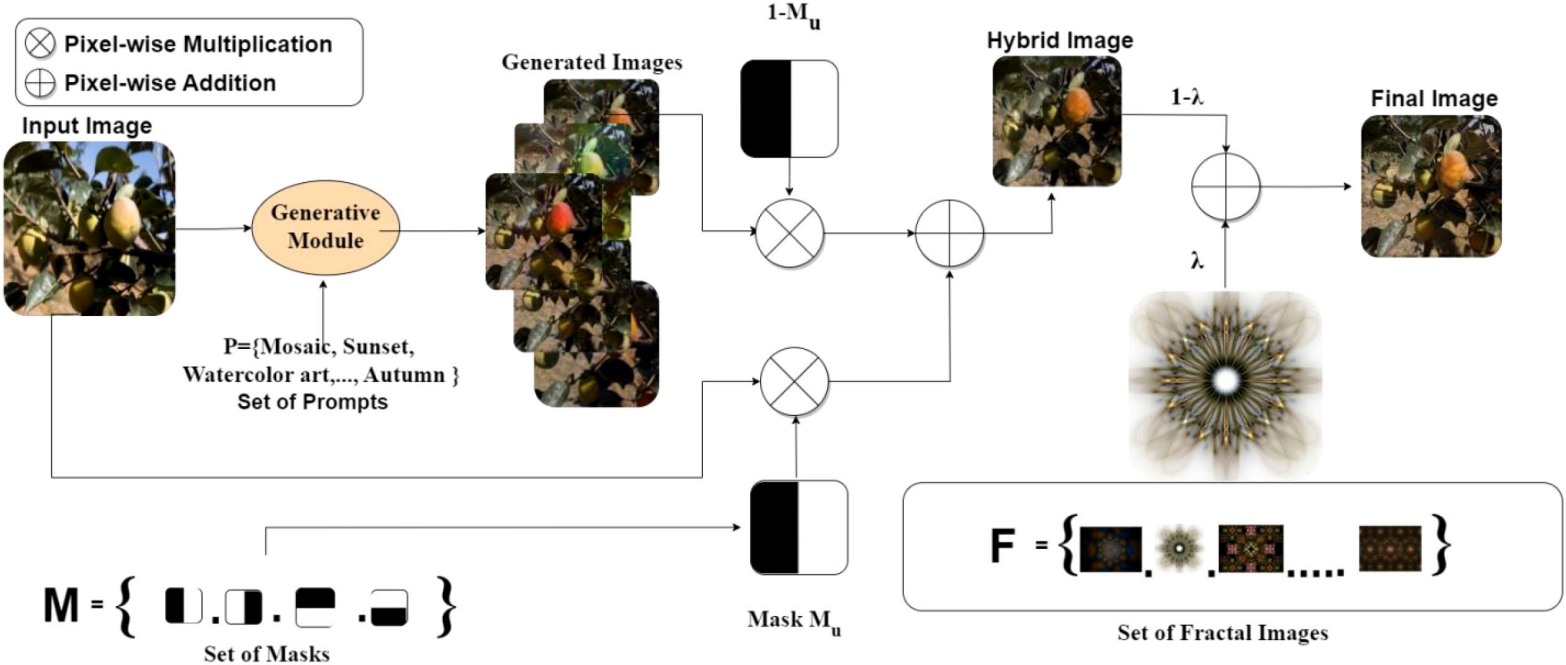
DiffuseMix framework diagram.

The masking mechanism *M*_u_ is then employed to achieve local stitching between the original image *I*_i_ and the generated variant *I*_ijvw_ (Wang H. et al., 2024), as illustrated in **Figure 2**. The mask explicitly specifies the source of each region—original or generated—thus preserving label integrity. As shown in Equation 2:

(2) $${\text{H}}_{ijvw}=({\hat{I}}_{ijvw}☉M_{u})+(I_{i}☉(1-M_{u}))$$

The final enhanced image A_ijvw_ is generated by blending the fractal image F_u_, where λ denotes the fusion coefficient. As shown in Equation 3:

(3) $${\text{A}}_{ijvw}=(1-\lambda )(I_{i}☉M_{u}+{\hat{I}}_{ijvw}☉(1-M_{u}))+\lambda F_{u}$$

Furthermore, DiffuseMix incorporates structured fractal patterns as controllable perturbations, enriching scene-layout and occlusion diversity while mitigating unnatural distortions in the synthesized content.

**Figure 3:**
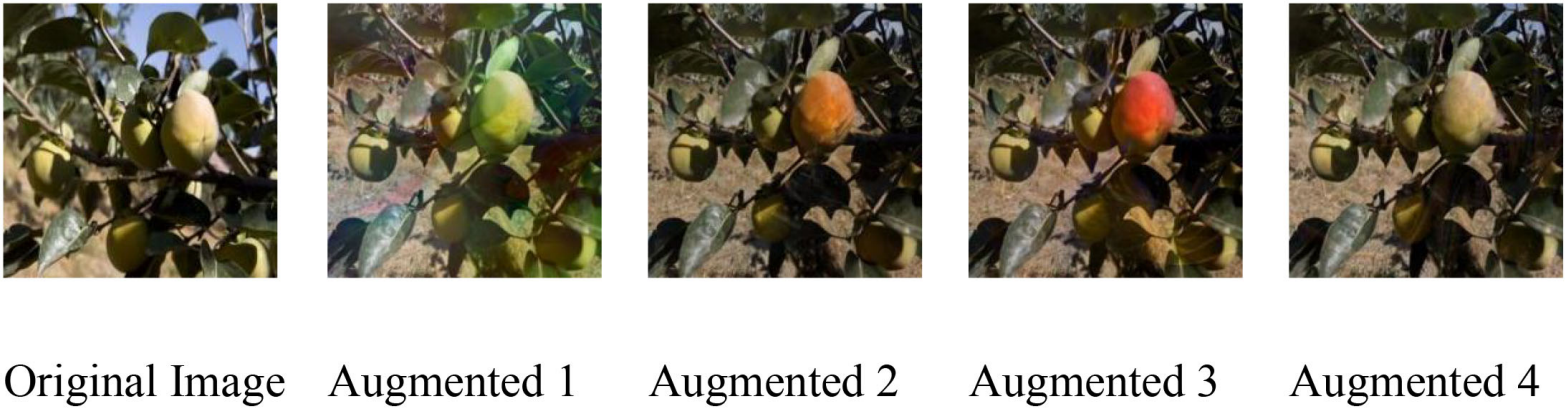
DiffuseMix processed image.

For the Taiqiu sweet persimmon dataset, raw images were curated and annotated with fruit–background semantic masks to preserve labeled fruit regions. A diffusion model was then fine-tuned on the original data to better capture fruit-specific characteristics and used to synthesize backgrounds with diverse lighting and textural conditions. Using the predefined masks, these backgrounds were selectively fused with the original fruit regions to ensure label consistency during blending. In addition, structured fractal patterns were introduced to simulate natural occlusions without covering the fruit. After quality inspection, the augmented samples were incorporated into the training set, increasing scene diversity and model robustness without compromising essential fruit features. Representative examples of the augmentation pipeline are shown in **Figure 3**.

#### 3.2.2 Data processing

After preprocessing, 3651 originals were retained and annotated. From these, 1857 images were selected via hierarchical sampling stratified by acquisition timestamps and then split into training and validation sets by random sampling at an 8:1.5 ratio, a partitioning that supports robust learning of complex feature representations while enabling hyperparameter tuning and overfitting monitoring. The remaining 1794 images were reserved as an independent held-out test set.

To increase data diversity, DiffuseMix was applied to the training and validation sets, tripling their sizes while leaving the test set unchanged. The final composition was: training, 4692 images (1,564 × 3); validation, 879 images (293 × 3); and test, 1794 images.Importantly, the held-out test set remained strictly unchanged to avoid data leakage and ensure a fair performance evaluation.

This structured dataset design ensures comprehensive coverage of fruit growth stages and environmental changes, facilitating the development of high-performance deep learning models for precision agriculture applications.

### 3.3 Research methodology

#### 3.3.1 YOLO11 detection model

YOLO11 is a state-of-the-art object-detection framework that balances accuracy and speed through structural refinements and lightweight optimization, making it suitable for real-time deployment in complex scenes. YOLO11 incorporates optimization strategies including adaptive anchor box computation, dynamic label assignment, and mixed precision training. Compared to YOLOv8’s static anchor boxes and YOLOv10’s simplified label assignment, it better adapts to the characteristics of the persimmon dataset—small sample size and high annotation noise—reducing overfitting risks while achieving faster training convergence.

Building upon YOLOv8, YOLO11 further refines the backbone network and feature fusion mechanism by introducing C3k2 dynamic structural units, which enables adaptive feature extraction through flexible convolutional paths, thereby balancing representational capacity and computational efficiency across diverse tasks (Zhu et al., 2025). Compared to the C2f module in YOLOv8 and the C2f-FL module in YOLOv10, this approach more effectively captures salient features such as color and shape in persimmons. In addition, YOLO11 incorporates an advanced attention mechanism within its feature pyramid: by jointly modeling channel-wise selection and spatial context, it suppresses background interference while emphasizing salient target features.

In the detection head, YOLO11 employs lightweight convolutional operators for reconstruction. Compared to YOLOv8 and YOLOv10, this approach not only reduces parameter size and computational complexity but also significantly improves both inference speed and detection accuracy (Zhao et al., 2025). Together with the preceding design enhancements, these refinements allow YOLO11 to sustain high operational efficiency and robust performance across multi-scale object detection, complex scene adaptation, and real-time deployment.

#### 3.3.2 YOLO11-FC2T network model construction

**Figure 4:**
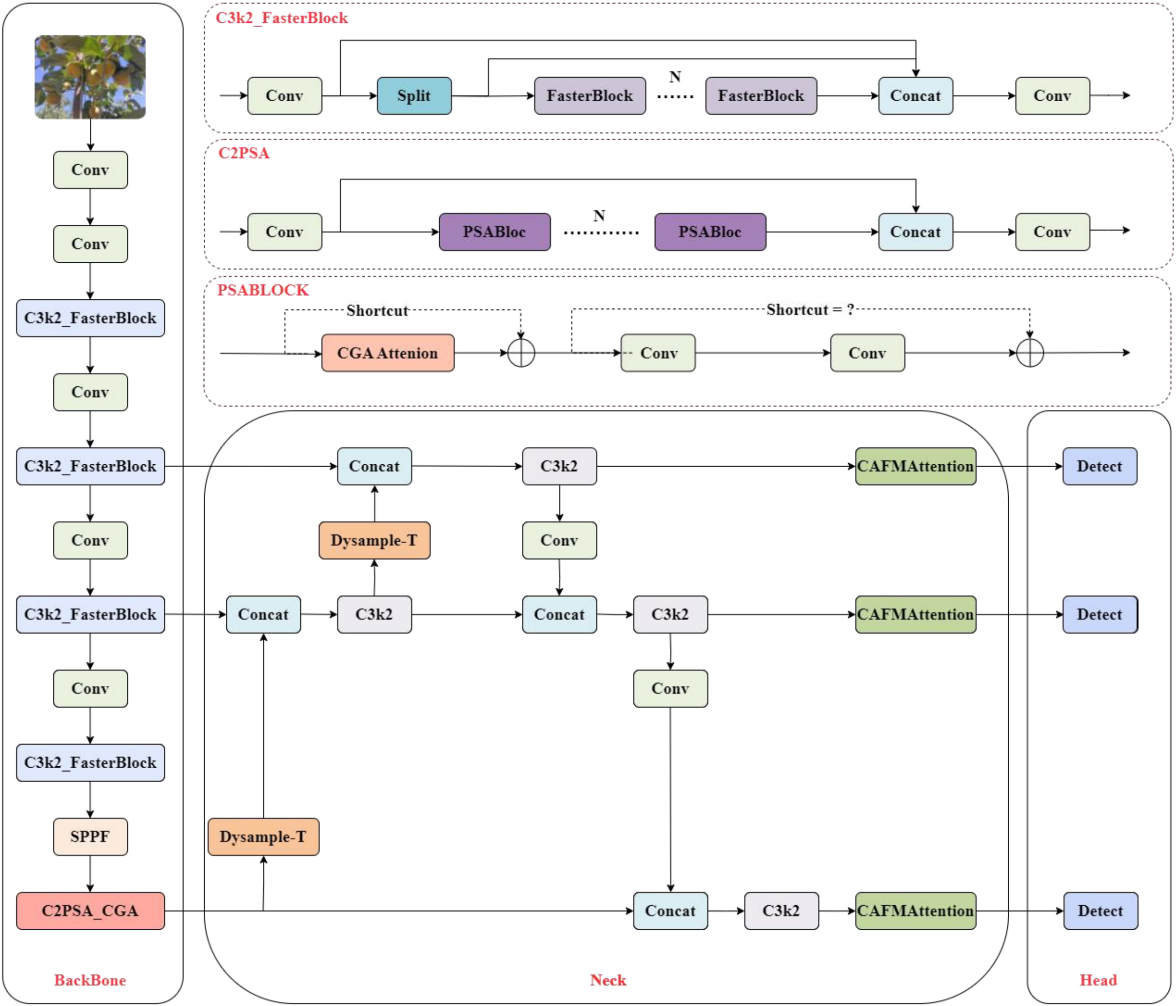
YOLO11-FC2T architecture diagram.

To enhance YOLO11 for detecting and counting Taiqiu sweet persimmons during the yellow-green color transition period, we propose YOLO11-FC2T—an efficient improvement framework for orchard scenarios whose architecture is shown in **Figure 4**. In the backbone, the C3k2 module is upgraded with FasterBlock and partial convolutions (PConv) to reduce redundant computation and memory access while improving the capture of fine-grained edges and subtle fruit–leaf chromatic differences. After feature extraction, Cascaded Group Attention (CGA) is introduced to increase feature diversity and capacity via block-wise, cross-head cascading, thereby enhancing discriminability without materially increasing computational cost. For the neck, we design a three-layer Dysample-T dynamic upsampling module, inspired by DySample, that adaptively adjusts upsampling weights based on feature content to improve the recall of small targets. In the deep fusion stage, the CAFM Attention module strengthens inter-layer information exchange and class separation through a combined convolution and attention mechanism, enabling accurate separation of similarly colored targets in complex backgrounds. Together, these components sustain high identification and counting accuracy during the transition period while controlling model complexity and meeting real-time deployment requirements in orchard settings.

#### 3.3.3 C3k2_FasterBlock

**Figure 5:**
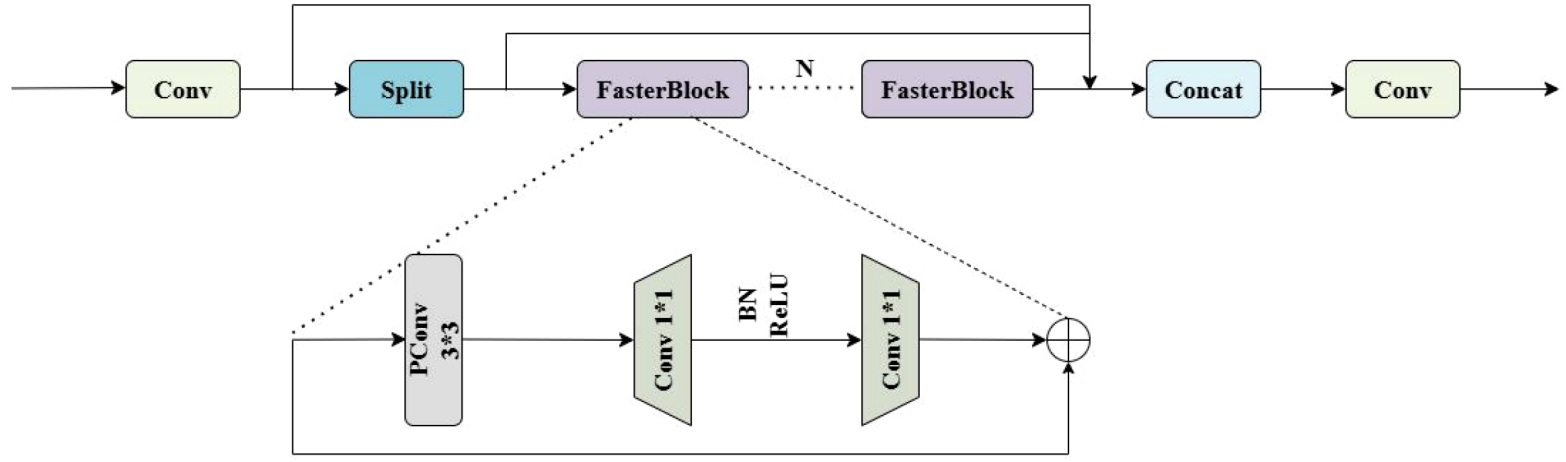
C3k2_fasterblock module architecture.

To address the practical challenges in detecting Taiqiu sweet persimmons during the yellow-green transition period—such as similar fruit-leaf colors, low contrast, frequent occlusion and adhesion, and a high proportion of small targets—this study replaces specific residual blocks in the C3 (CSP) module of the YOLO11 backbone network with FasterBlock (Wang J. et al., 2024), the overall design is shown in **Figure 5**. This improvement enhances real-time performance and deployment efficiency while maintaining accuracy. By incorporating partial convolution (PConv), redundant computations and memory access are further reduced, increasing feature extraction throughput. Under conditions of weak illumination, dappled lighting, and interference from branches and leaves, the model generates clearer and more stable intermediate features. The enhanced detector significantly improves the recall rate for early-color-change fruits and distant small targets while reducing the false detection rate. The implementation preserves the original multi-scale architecture and detection heads, replacing only parts of the main branch in the C3 module. This results in reduced Giga floating-point operations per second(GFLOPs), increased frames per Second(FPS), and improved compatibility with edge devices, making the model particularly suitable for orchard applications such as pre-harvest inspection and dynamic yield estimation.

The C3 module initially splits the input features into a main branch and a shortcut branch. The main branch stacks N FasterBlock units, each of which first applies a 3×3 partial convolution (PConv) to obtain low-cost base feature responses, followed by two sequential 1×1 pointwise convolutions. Batch normalization and ReLU activation are inserted exclusively between these two pointwise convolutions, reducing computational overhead while maintaining feature diversity. Each block incorporates skip connections to ensure training stability and preserve original feature information. The shortcut branch remains unchanged and is concatenated with the processed main branch features. Finally, a 1×1 convolution performs channel alignment and semantic integration, producing refined features for subsequent detection heads.

This architecture achieves an optimization of efficiency and representation quality. The PConv operation reduces computational costs by decreasing memory access and floating-point operations (GFLOPs), while the streamlined normalization-activation scheme minimizes processing latency. Identity mappings through residual connections preserve fine-grained spatial details and enhance gradient flow during optimization. These design choices collectively generate more discriminative intermediate features and improve processing throughput in challenging scenarios involving similar-colored backgrounds, low contrast, occlusions, adhesion, and dense small or distant targets. The resulting feature representations provide robust support for accurate real-time identification and counting of Taiqiu sweet persimmons.

#### 3.3.4 C2PSA_CGA

**Figure 6:**
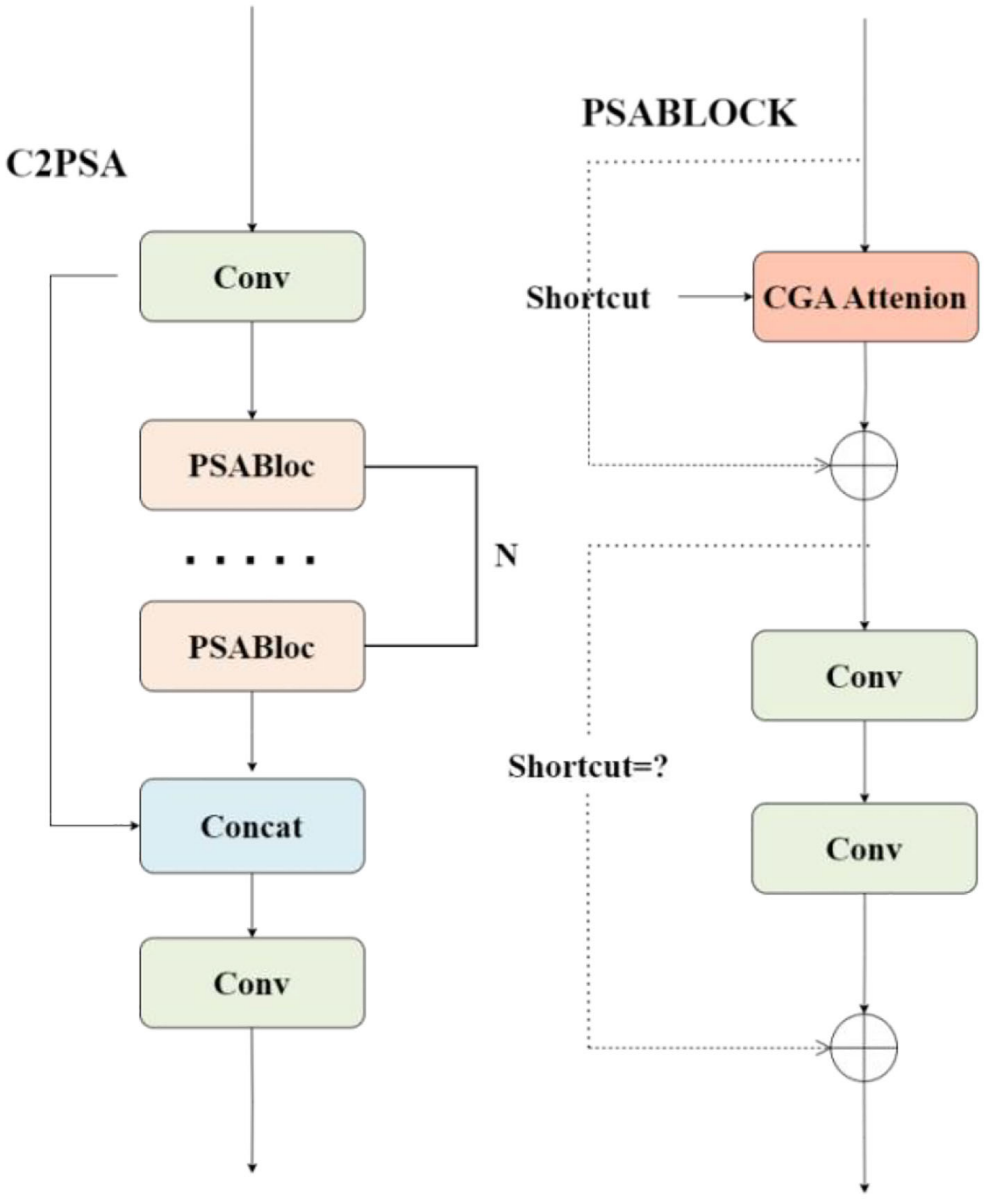
C2PSA_CGA module structure diagram.

Although C3k2_FasterBlock improves feature-extraction efficiency, its single-path design is insufficient for modeling the collective characteristics of densely clustered Taiqiu sweet persimmons in complex orchard scenes. In particular, it struggles to capture inter-fruit spatial relationships and occlusion patterns, leading to counting errors for tightly arranged or partially occluded specimens (Dorj et al., 2017). To remedy this, we introduce a feature-enhancement module based on Cascaded Group Attention (CGA) (Wang J. et al., 2025), which applies cascaded, group-wise attention to achieve precise multi-scale feature focusing and stronger context modeling; the overall design is shown in **Figure 6**.

**Figure 7:**
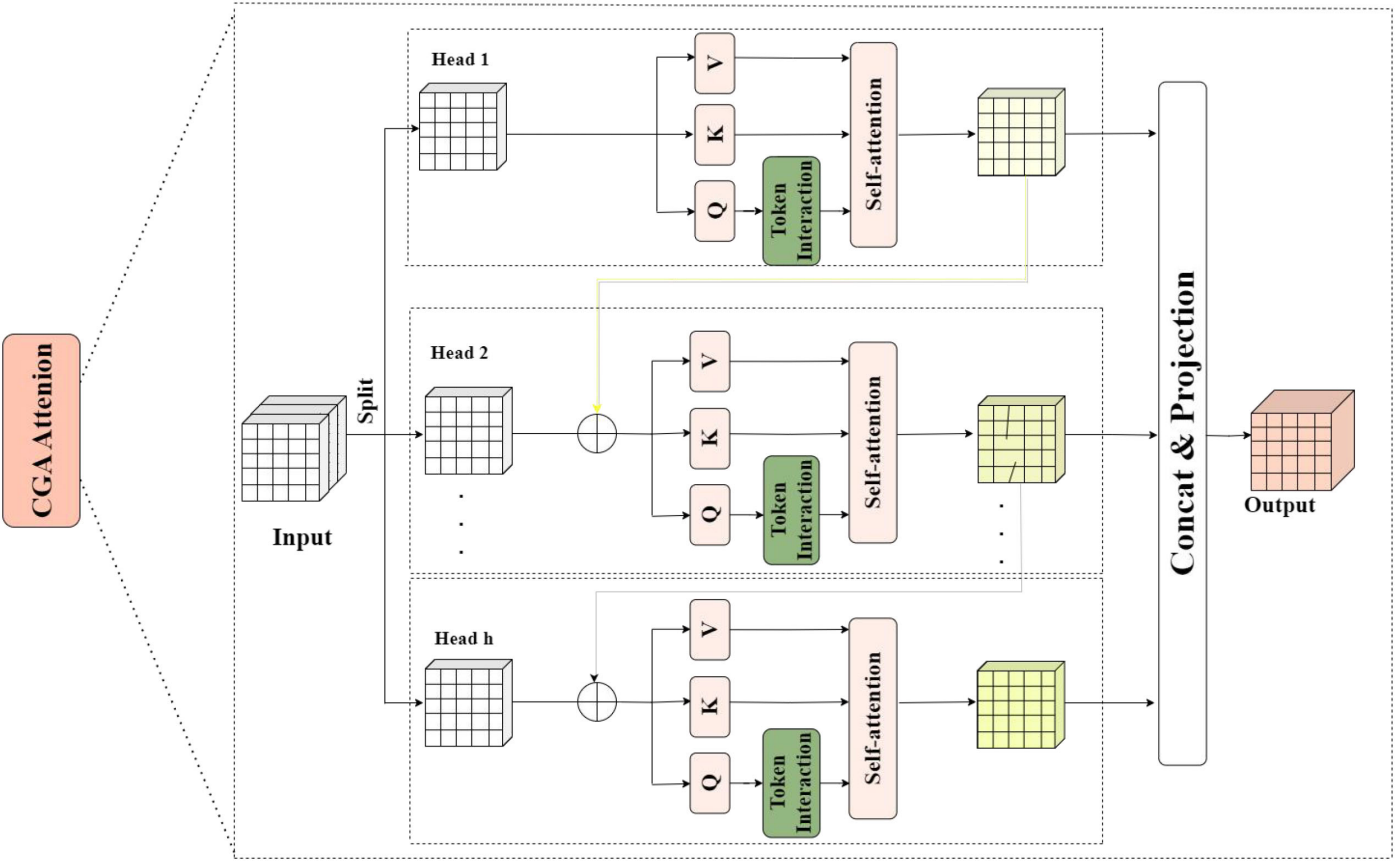
CGA module structure diagram.

To address the loss of contextual information and counting inaccuracies during the yellow-green transition period of Taiqiu sweet persimmons—challenges arising from similar-colored backgrounds, partial occlusions, and fruit adhesion—this study replaces the original C2PSA units in the higher layers of the backbone network with the C2PSA_CGA module, which incorporates Cascaded Group Attention (CGA). The CGA structure diagram is shown in **Figure 7**, the module begins by splitting the input features along channel and group dimensions into h distinct subsets, each processed by a dedicated attention head.

For the j-th group feature X_ij_, the Q, K, and V values are obtained through linear mapping, and the attention response is calculated. The output of attention is shown in Equation 4:

(4) $${\bar{\text{X}}}_{ij}=Attn(X_{ij}W_{ij}^{Q},X_{ij}W_{ij}^{K},X_{ij}W_{ij}^{V})$$

W_ij_^Q^, W_ij_^K^,and W_ij_^V^ denote the projection matrices corresponding to j attention heads respectively.

The output features from each attention head are then fused via a concatenation operation and restored to the original channel dimension through a linear mapping. As shown in Equation 5:

(5) $${\bar{\text{X}}}_{i+1}=Concat{\left[\bar{X_{ij}}\right]}_{j=1:h}W_{i}^{P}$$

In contrast to the parallel and independent structure of conventional Multi-Head Self-Attention (MHSA), C2PSA_CGA employs a cascade strategy. This allows the j-th attention head to incorporate the output information of the j-1-th attention head during computation, thereby establishing sequential dependencies. This approach explicitly models the association and occlusion propagation patterns among fruit clusters. The cascade strategy is expressed as shown in Equation 6:

(6) $$X_{\text{i}j}^{'}=X_{ij}+{\bar{X}}_{i(j-1),}\;1<j\leq h$$

The outputs of all heads are then integrated through concatenation and projection (using linear or 1×1 convolutional fusion). Combined with a multi-branch convolutional residual structure, the module strengthens the representation of associations among dense targets while maintaining computational efficiency. This results in significantly improved accuracy and stability in fruit identification and counting under the challenging conditions of the color transition stage.

The PSABLOCK structure integrates three parallel processing branches: a Cascaded Group Attention (CGA) branch, a 1×1 convolutional branch with residual connection, and a 3×3 convolutional branch with residual connection. The outputs of these branches are aggregated through element-wise summation at the terminal stage. This design significantly enhances the diversity of feature representations and improves background suppression capability without introducing additional computational overhead at the block level. Consequently, the module demonstrates superior adaptability to complex scenarios in Taiqiu persimmon orchards, particularly under conditions of low contrast and significant occlusion or fruit clumping.

The synergistic interaction between the C2PSA_CGA and C3k2_FasterBlock modules enhances the model’s capacity to represent features of densely clustered Taiqiu sweet persimmons in complex orchard environments. This improvement establishes a more discriminative feature basis for subsequent identification and counting tasks, thereby effectively mitigating counting inaccuracies resulting from occlusion and dense spatial arrangements.

#### 3.3.5 Dysample_T

**Figure 8:**
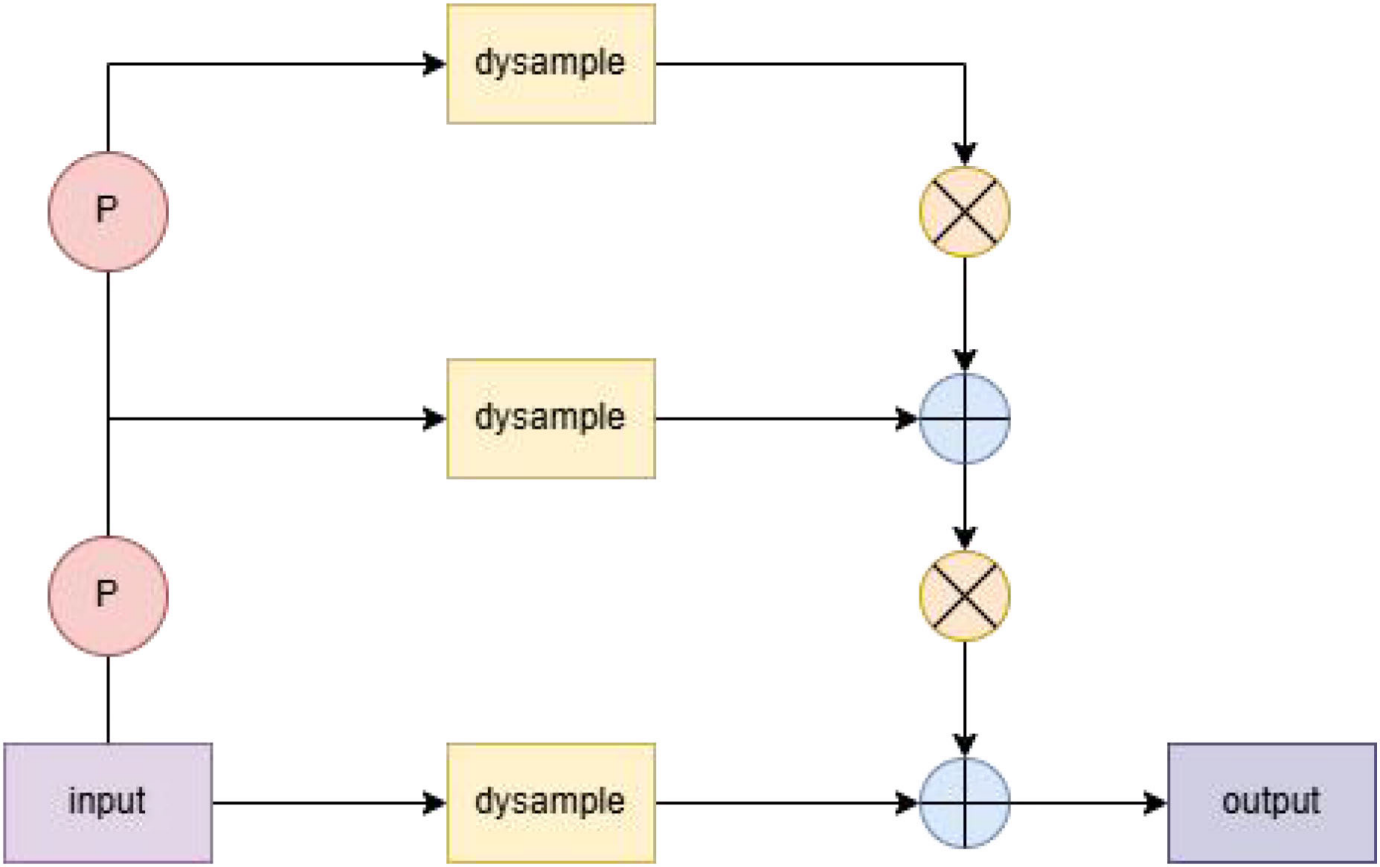
Dysample_T module architecture.

To accommodate the scale variations and intricate texture characteristics of Taiqiu sweet persimmons in natural harvesting environments, this study draws upon the DySample concept (Wang Q. et al., 2025) to design a multi-scale upsampling module named DySample_T within the YOLO neck network. The module structure is illustrated in **Figure 8**. Its design focuses on three core principles: dynamic multi-scale sampling, cross-scale feature aggregation, and fine-grained texture enhancement. Through a three-stage dynamic upsampling and fusion mechanism, the module effectively integrates semantic information with detailed feature representations.

The module first performs three distinct dynamic upsampling operations on the input features. Each operation upscales features by a predetermined factor, generates scale-specific sampling offsets through independent offset learners, and constructs dynamic sampling coordinates by combining these offsets with a predefined coordinate grid. The sampling process employs a channel-grouping strategy wherein input features are divided into groups prior to applying bilinear interpolation. This approach preserves fine-grained textures while enabling adaptive adjustment to feature distribution variations through dynamic offsets. Subsequently, the three upsampled feature sets are unified to the largest target scale, with spatial consistency maintained through additional bilinear interpolation. The concatenated multi-scale features then undergo channel-wise integration via a fusion convolutional layer, facilitating complementary information exchange across scales. The dynamic offset mechanism allows sampling weights to adaptively evolve according to feature characteristics, thereby mitigating the detail loss typically associated with fixed interpolation methods. Furthermore, the grouped sampling strategy enhances local texture capture capability, making it particularly effective for identifying subtle chromatic differences characteristic of Taiqiu sweet persimmons during the yellow-green transition period.

Compared with conventional upsampling methods, the proposed design demonstrates three principal advantages: (1) The three-stage differential sampling strategy establishes denser inter-scale connections, thereby enhancing the detection sensitivity for small and distant fruits; (2) The dynamic offset and grouping mechanisms collectively improve the preservation of fine textural details, which is particularly beneficial for distinguishing targets from backgrounds in low-color-contrast scenarios; (3) The multi-scale fusion process progressively integrates semantic information with fine-grained features, effectively reducing the information loss typically associated with single-step large-scale upsampling while simultaneously improving both detection recall rate and boundary localization precision.

Within the YOLO architecture, the output features of DySample_T are directly integrated into the neck feature pyramid (P3/P4/P5), It provides a more discriminative multi-scale feature representation for persimmon detection. This enhancement ensures robust detection performance, particularly under challenging conditions including strong illumination, partial occlusion, and dense fruit arrangements.

#### 3.3.6 CAFMAttention

**Figure 9:**
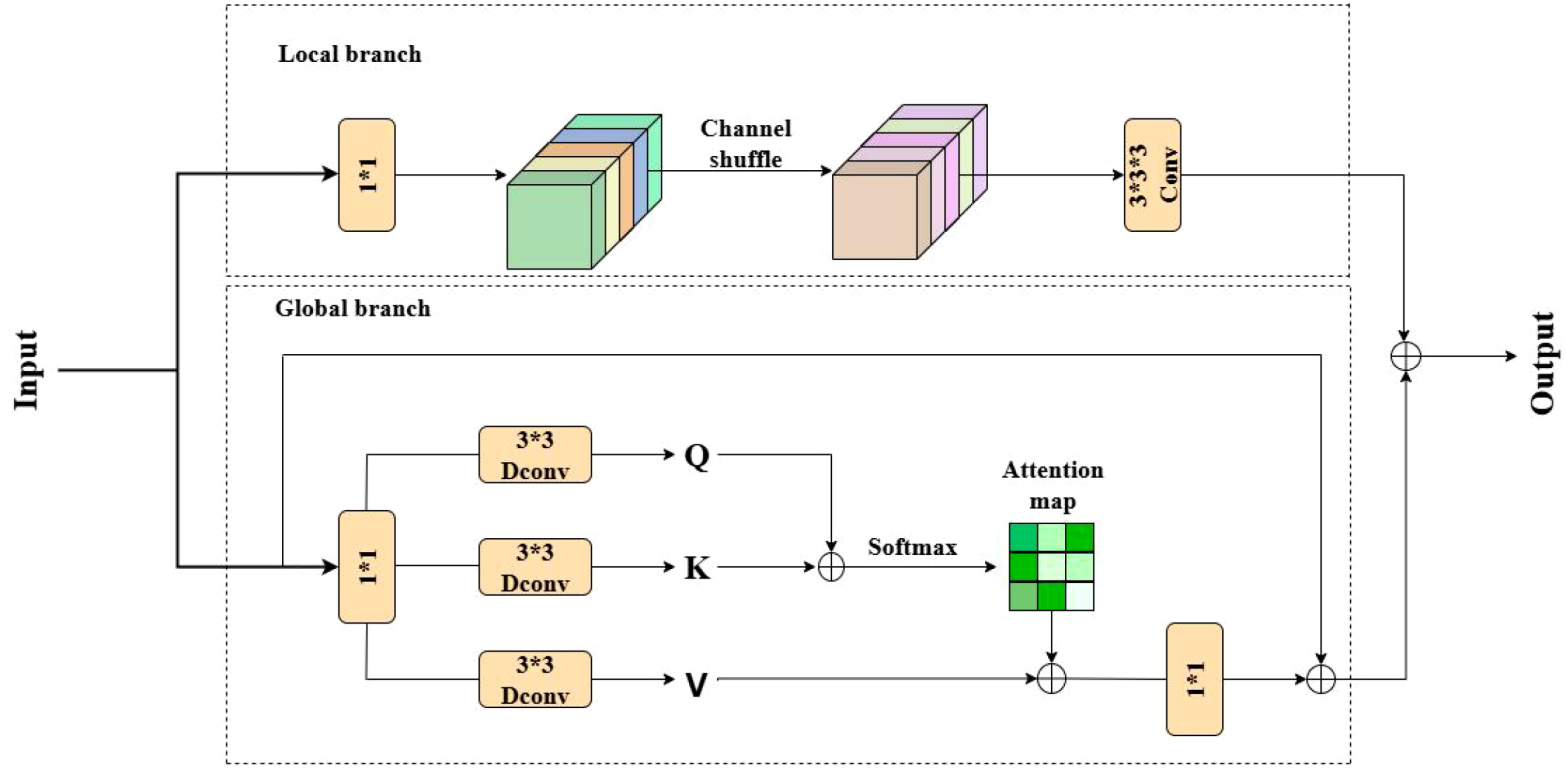
CAFM attention module architecture.

Orchard scenes during the color-transition typically feature near-color backgrounds, uneven illumination, partial occlusion, and fruit adhesion, so fine textures such as fruit edges and stems are easily lost during upsampling and multi-scale fusion, leading to false positives (e.g., leaves misclassified as fruit) and false negatives (e.g., missed adhered fruit) (Ning et al., 2022). To enhance feature discriminability while controlling parameters, we insert the CAFMAttention module (Yao et al., 2025) before the fusion and upsampling layers in the neck (**Figure 9**). CAFMAttention comprises parallel Local and Global branches whose outputs are merged by residual summation: the Local branch preserves fine details while suppressing noise, whereas the Global branch aggregates context and attenuates background interference. Practice shows, CAFMAttention more clearly highlights, edges, calyxes, and specular reflections of Taiqiu sweet persimmons under low-light, near-color conditions, thereby reducing misdetections and improving counting consistency. The module preserves resolution and interface compatibility with the original PAN structure, enabling efficient deployment on edge devices.

The Local branch adopts a sequential (1x1) convolution → Channel Shuffle → (3x3) convolution block. The initial (1x1) layer reduces channels and reorders features, the Channel Shuffle operation facilitates cross-channel information flow, and the terminal (3x3) layer sharpens fine-grained textures—such as fruit edges, stems, and specular highlights. The block output is then fused with the input via a residual connection to preserve locality and stabilize optimization. The local branch can be formulated as Equation 7:

(7) $$F_{conv}=W_{3\times 3\times 3}(CS(W_{1\times 1}(Y)))$$

where F_conv_ is the output of local branch, W_1×1_ denotes 1 × 1 convolution, W_3×3×3_ denotes 3 × 3 × 3 convolution, CS() represents channel shuffle operation, and Y is the input feature.

The process of the Global branch is as follows: First, it reduces the dimension through a 1×1 convolution to obtain an intermediate feature vector that integrates the information of query (Q), key (K), and value (V). The mixed features are then split along the channel dimension into three independent features, which are processed in parallel through three 3×3 depthwise separable convolution branches to generate $\hat{Q},\hat{K},\hat{V}$ respectively. An attention map is generated through Softmax (QK^T^), and V is weighted and aggregated. After that, the channel dimension is restored through a 1×1 convolution linear projection, and a residual connection is made with the input feature. The output F_att_ of global branch is defined as Equation 8, the output of Attention is shown in Equation 9:

(8) $${\text{F}}_{att}=W_{1\times 1}Attention(\hat{Q},\hat{K},\hat{V})+Y$$

(9) $$\text{Attention}(\hat{Q},\hat{K},\hat{V})=\hat{V}Soft\max(\hat{K}\hat{Q}/\alpha )$$

Following their respective residual connections, the outputs from both branches are fused at the module’s final stage. As shown in Equation 10:

(10) $${\text{F}}_{out}={\text{F}}_{att}+{\text{F}}_{conv}$$

The resulting features are then propagated to subsequent upsampling or fusion layers. The proposed architecture intentionally avoids incorporating grouped multi-head mechanisms or cascaded attention heads, strictly adhering to a “dual-branch + attention map + dual residual connection” paradigm. This design significantly reduces detection errors, such as misclassifying yellow leaves as fruits, and improves counting consistency, all while maintaining compatibility with the original backbone network and Path Aggregation Network (PAN) topology.

## 4 Experimental design

### 4.1 Experimental environment and parameter settings

The experimental platform and environment are detailed in the **Table 1**.

**Table 1:** Experimental environment configuration.

Configuration name	Version & model
GPU Model & Quantity	NVIDIA GeForce RTX 4090D
CPU Model	Intel^®^ Xeon^®^ Platinum 8270 CPU @ 2.70GHz
System RAM	128.0GB
Operating System	Windows 11 Home Chinese Edition (22H2 build)
Deep Learning Framework	torch2.2.0+cu118
CUDA with cuDNN version	CUDA 11.8 + cuDNN 8.9.7
Programming Languages and Dependency Libraries	timm == 0.9.12, mmcv-full == 2.2.0, numpy == 1.26.3

### 4.2 Training parameters

Key training parameters are summarized in **Table 2**. The training strategy adopts a learning rate warm-up mechanism, wherein the learning rate increases linearly from an initial value of 10% to the full preset value over the first three epochs. This approach mitigates potential gradient instability caused by random weight initialization during the initial training phase and facilitates stable acquisition of fine-grained fruit characteristics, such as the subtle color transition at the yellow-green boundary and surface spot patterns.

**Table 2:** Parameter setting.

Parameter name	Parameter value	Parameter name	Parameter value
epoch	230	patience	70
batch	16	amp	False
workers	16	iou	0.7
optimizer	SGD	lr0	0.01
size	640	lrf	0.01

## 5 Result and discussion

### 5.1 Comparison of detection performance among different models

To evaluate the performance of the proposed YOLO11-FC2T model, comparative experiments were conducted on the Taiqiu sweet persimmon dataset against seven representative object detection models: YOLOv5, YOLOv6, YOLOv8, YOLOv9, YOLO10, RT_DETR and YOLO11. The assessment specifically focused on each model’s capability in detecting Taiqiu sweet persimmons during the yellow-green color transition period. The quantitative results are presented in **Table 3**.

**Table 3:** Detection performance of different models.

Name	P	R	F1	mAP@0.5	mAP@0.5:0.95	FPS	GFLOPs
YOLOv5	0.903	0. 843	0.872	0.930	0.772	109.5	5.8
YOLOv6	0.902	0. 842	0.871	0.933	0.779	131.8	11.5
YOLOv8	0.899	0. 843	0.870	0.927	0.775	122.5	6.8
YOLOv9	0.892	0. 843	0.867	0.935	0.781	49.6	7.6
YOLO10	0.901	0. 832	0.865	0.924	0.764	83.5	8.2
RT_DETR	0.858	0.802	0.829	0.895	0.726	103.4	17.4
YOLO11	0.907	0. 839	0.872	0.932	0.772	84.0	6.2
YOLO11-FC2T	0.917	0. 867	0.891	0.948	0.812	60.5	7.0

We evaluate performance using multi-dimensional metrics. First, the F1 score—computed from precision (P) and recall (R)—provides a balanced assessment of accuracy and completeness. Second, mAP@0.5 (mean average precision at IoU = 0.5) and mAP@0.5-0.95 (mean average precision averaged over IoU thresholds from 0.50 to 0.95 in 0.05 increments) quantify detection quality across overlap conditions. Finally, GFLOPs (giga floating-point operations) and FPS (frames per second) measure computational complexity and inference speed, respectively, thereby indicating computational cost and deployment readiness.

Experimental results demonstrate that YOLO11-FC2T achieves superior performance across all detection accuracy metrics while maintaining a favorable balance in computational efficiency. Specifically, YOLO11-FC2T attained the highest scores in precision (91.7%), recall (86.7%), and F1-score (89.1%) among all evaluated models, representing improvements of 1.0%, 2.8%, and 1.9%, respectively, over the baseline YOLO11. More notably, mAP@0.5 increased by 1.6%, while mAP@0.5:0.95 showed a substantial improvement of 4.0%. The proposed model significantly outperforms other comparative models such as YOLOv9, demonstrating enhanced capability in identifying fruits against low-contrast backgrounds during the color transition period.

Regarding computational efficiency, although YOLO11-FC2T’s GFLOPs increased by approximately 12.9% compared to YOLO11, it maintains lower computational complexity than both YOLOv6 and YOLO10. While the frames per second (FPS) decreased by approximately 27.9% relative to YOLO11, the achieved processing speed is still higher than YOLOv9. Crucially, YOLO11-FC2T achieves superior mAP@0.5:0.95 performance at similar inference speeds while still satisfying the real-time detection requirements for orchard applications (FPS ≥30). In addition to comparing the models of the YOLO series, we also compared them with the RT-DETR model. The results showed that the performance of other YOLO series models was superior to that of the RT-DETR model. Therefore, YOLO11-FC2T achieves a perfect balance between accuracy and robustness for detecting Taiqiu sweet persimmons during the color transition period without compromising practical deployability, establishing it as the optimal approach for balancing accuracy and efficiency in this specific task.

### 5.2 Ablation studies

In the ablation study, each proposed module (A: C3k2_FasterBlock, B: C2PSA_CGA, C: CAFMAttention, D: DySample_T) was incrementally integrated into the baseline model to evaluate their individual and synergistic effects on performance (**Table 4**). Experimental groups 16 utilized conventional data augmentation methods, whereas Group 1–15 and 17 employed the DiffuseMix augmentation strategy.

**Table 4:** Ablation experiment results.

Model	Module				P	R	F1	mAP@0.5	mAP@0.5:0.95	FPS	GFLOPs
	A	B	C	D							
1					0.907	0.839	0.872	0.932	0.772	84.0	6.2
2	✓				0.884	0.865	0.874	0.932	0.768	84.5	6.2
3		✓			0.907	0.845	0.875	0.931	0.779	70.6	7.1
4			✓		0.896	0.855	0.875	0.928	0.774	80.2	7.1
5				✓	0.888	0.865	0.876	0.932	0.783	66.7	7.0
6	✓	✓			0.899	0.866	0.882	0.937	0.794	74.8	6.2
7	✓		✓		0.907	0.856	0.881	0.934	0.792	59.6	6.3
8			✓	✓	0.907	0.863	0.884	0.938	0.799	60.6	7.1
9		✓	✓		0.911	0.855	0.882	0.941	0.804	55.6	6.2
10		✓		✓	0.888	0.877	0.882	0.942	0.801	75.2	7.0
11	✓			✓	0.908	0.865	0.886	0.938	0.797	70.5	7.1
12	✓	✓	✓		0.922	0.853	0.886	0.943	0.806	54.6	6.2
13	✓	✓		✓	0.913	0.861	0.886	0.939	0.809	50.6	7.0
14		✓	✓	✓	0.921	0.858	0.888	0.944	0.808	57.3	7.0
15	✓		✓	✓	0.914	0.861	0.887	0.944	0.805	54.1	7.1
16	✓	✓	✓	✓	0.913	0.856	0.883	0.943	0.812	63.5	7.0
17	✓	✓	✓	✓	0.917	0.867	0.891	0.948	0.812	60.5	7.0

As shown in **Table 4**, a ‘√’ in the table indicates the model is included in the baseline model, while a blank indicates it is not.The results show that introducing either module A or B alone consistently increases precision and mAP with negligible impact on GFLOPs and FPS. And module A’s replacement scheme slightly lowers computational complexity and yields a modest gain in inference throughput. Incorporating module C in isolation markedly improves recall and mAP@0.5:0.95—especially for occluded clusters and low-light cases—but at the cost of higher computational load and reduced speed. By contrast, adding module D alone offers limited gains in recall and overall mAP yet proves effective for small and distant targets, while maintaining near-baseline GFLOPs and FPS.

For module combinations, jointly integrating A and B yields larger gains in precision (P), mAP@0.5, and mAP@0.5-0.95, with GFLOPs stable or slightly reduced and FPS increased, confirming the effectiveness of the “efficient feature extraction + cross-head semantic transmission” mechanism under near-color occlusion. Combining C and D substantially improves recall and mAP@0.5-0.95 but increases GFLOPs and lowers FPS, indicating extra overhead during feature fusion and reconstruction. The full model integrating all four modules delivers the best performance across all metrics; although computational complexity rises moderately and inference speed declines slightly, the trade-off is justified by clear core benefits—markedly reducing the misclassification of yellow leaves as fruit during the color-transition and improving counting stability. Overall, the ablation results validate both the individual contributions and the synergistic effects of the modules in enhancing detection and counting performance.

### 5.3 Causal analysis comparison

In Section 5.2, this paper validates the improvement effects of each structural improvement module and their combinations on model performance through systematic ablation experiments, and preliminarily observes the differentiated contributions of different modules in terms of precision, recall, and multi-scale detection capabilities. However, the results of ablation experiments may still be influenced by factors such as random initialization and training process fluctuations. It is difficult to comprehensively reflect the stability and repeatability of performance improvements of each module based on a single experimental value alone.

Therefore,To eliminate the interference caused by training randomness and provide more reliable evidence, we introduce a causal analysis perspective, where the performance change relative to the baseline model (ΔmAP@0.5) is regarded as the estimated Average Treatment Effect (ATE) under a paired evaluation setting. Meanwhile, to comprehensively assess the significance, magnitude, and robustness of performance improvements, four metrics are reported:To eliminate the interference caused by training randomness and provide more reliable evidence, we introduce a causal analysis perspective, where the performance change relative to the baseline model (ΔmAP@0.5) is regarded as the estimated Average Treatment Effect (ATE) under a paired evaluation setting. Meanwhile, to comprehensively assess the significance, magnitude, and robustness of performance improvements, four metrics are reported:

1. mAP@0.5 (Mean ± Std) — measuring training stability;
2. ΔmAP (ATE) — quantifying improvement magnitude;
3. p-value — testing whether improvements are statistically superior to random fluctuations;
4. 95% CI of ΔmAP — depicting the uncertainty range of performance changes.

In terms of statistical testing, this paper employed the paired t-test to evaluate the performance differences between each model configuration and the baseline model. This testing method can control the influence of random initialization and training fluctuations, and directly compare the performance differences of different models in the corresponding repeated experiments, thereby more accurately reflecting the actual impact of structural changes or strategy introduction. The experimental results are shown in **Table 5**, where A = C3k2_FasterBlock, B = C2PSA_CGA, C = CAFMAttention, and D = Dysample_T, whereas Group 1–10 and 12 employed the DiffuseMix augmentation strategy,Group 11 employed the original enhancement method.

**Table 5:** Significance and robustness verification results.

Model	Module				mAP@0.5(Mean ± Std)	ΔmAP vs base	p-value	95%CI of ΔmAP
	A	B	C	D				
1					0.932 ± 0.002	–	–	–
2	✓				0.933 ± 0.001	+0.001	≈0.42	[-0.001,+0.003]
3		✓			0.932 ± 0.002	+0.000	≈0.78	[-0.002,+0.002]
4			✓		0.928 ± 0.001	-0.004	≈0.03	[-0.006,-0.001]
5				✓	0.933 ± 0.001	+0.001	≈0.18	[-0.001,+0.003]
6	✓	✓			0.938 ± 0.002	+0.007	<0.01	[+0.003,+0.010]
7	✓			✓	0.939 ± 0.001	+0.007	<0.01	[+0.005,+0.010]
8			✓	✓	0.938 ± 0.002	+0.006	<0.01	[+0.003,+0.009]
9		✓	✓	✓	0.943 ± 0.001	+0.011	<0.001	[+0.009,+0.014]
10	✓		✓	✓	0.944 ± 0.001	+0.012	<0.001	[+0.010,+0.015]
11	✓	✓	✓	✓	0.943 ± 0.001	+0.011	<0.01	[+0.008,+0.014]
12	✓	✓	✓	✓	0.948 ± 0.001	+0.016	<0.001	[+0.013,+0.019]

The experimental results show that when only the C3k2_FasterBlock, C2PSA_CGA, or Dysample_T module is introduced, the performance change of the model is limited and does not show stable statistical significance. Among them, when CAFMAttention is introduced alone, the model shows a performance decline trend in multiple repeated experiments. In contrast, the multi-module combined configuration achieves consistent and significant performance improvement on mAP@0.5. The corresponding performance gain is consistent in different repeated experiments, and the confidence interval is completely within the positive range, indicating that these improvements have good statistical reliability.

Under the complete model structure including C3k2_FasterBlock, C2PSA_CGA, CAFMAttention, and Dysample_T, the model trained using traditional data augmentation methods is significantly better than the baseline model. While keeping the model structure unchanged, introducing the DiffuseMix data augmentation strategy further improves the model performance and achieves the optimal performance on the mAP@0.5 indicator. This result indicates that the multi-module collaboration at the structural level and the data-level enhancement strategy have complementary effects in performance improvement.

Overall, the above experimental results show that the proposed method exhibits a consistent performance improvement trend in multiple independent repeated experiments, indicating that the observed performance gain is not caused by random training fluctuations, but rather by the synergy effect of the proposed module combination and the DiffuseMix data augmentation strategy.

In conclusion, based on the average treatment effect causal analysis, the results of the ablation experiments in Section 5.2 have been supplemented and verified from the statistical perspective. It not only quantifies the average performance contribution of each module but also reveals the synergistic gain relationship between different structural improvements. The analysis results are highly consistent with the performance change trends observed in the ablation experiments, further enhancing the stability and interpretability of the proposed improvement scheme in the complex scene of the yellow-green transition period of the sweet persimmon.

### 5.4 Model efficiency evaluation

Although detection accuracy is essential, model complexity must also be considered to ensure practical deployability in real-world orchard scenarios. To this end, we evaluate the computational cost of the baseline YOLO11 and the proposed YOLO11-FC2T in terms of the number of parameters (Params, M) and per-image inference latency (ms). The measurements were conducted on a single NVIDIA RTX 4090D GPU with an input size of 640×640 and batch size = 1, ensuring a fair comparison under identical conditions. The results are summarized in **Table 6**.

**Table 6:** Model complexity comparison.

Model	Params (M)	Change	Latency (ms)	Change
YOLO11	2.59	–	15.06	–
YOLO11-FC2T	2.95	+13.9%	19.04	+26.4%

The baseline YOLO11 has 2.59 M parameters and a latency of 15.06 ms per image. Despite introducing multiple attention-enhanced structural components, YOLO11-FC2T only slightly increases the parameters to 2.95 M and the latency to 19.04 ms (≈26.4% overhead). Overall, the proposed model achieves remarkable improvements in mAP while maintaining lightweight characteristics and high inference efficiency.

These results demonstrate that the proposed YOLO11-FC2T strikes a favorable balance between accuracy and efficiency, and is suitable for deployment on edge computing devices in precision agriculture applications.

### 5.5 Visualization of detection results

**Figure 10:**
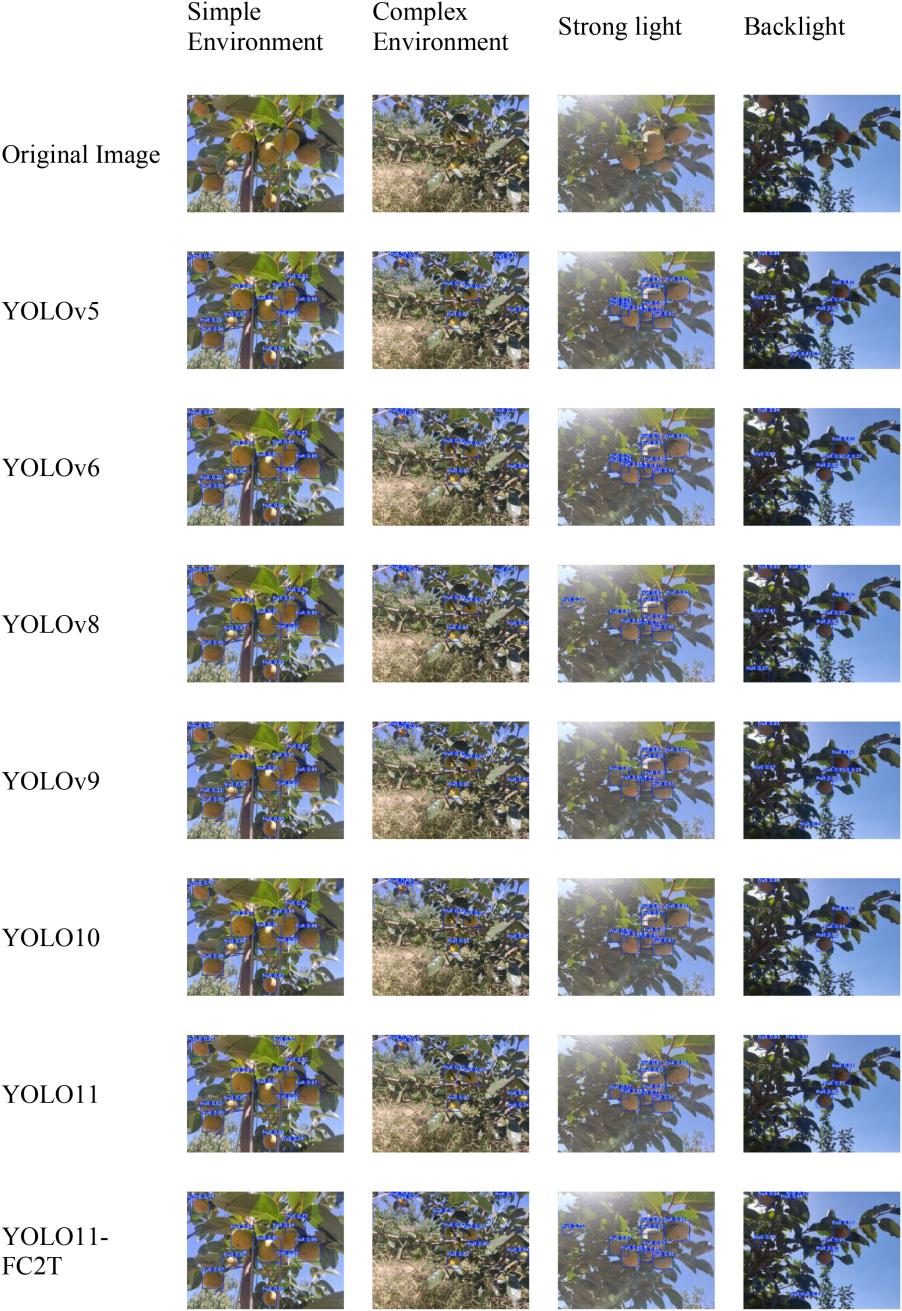
Performance of different models in different environments.

To assess performance under real orchard conditions, **Figure 10** visualizes detections from YOLO11-FC2T and six baselines (YOLOv5, YOLOv6, YOLOv8, YOLOv9, YOLO10, YOLO11) across diverse environments during the color-transition of Taiqiu sweet persimmons. Qualitative comparisons indicate that YOLO11-FC2T yields more accurate and stable results across all tested scenarios—improving localization and recall for small or partially occluded fruit, reducing false positives in near-color backgrounds, and suppressing artifacts such as fragmented boxes and duplicated detections—thereby outperforming the baselines in complex lighting and occlusion conditions.

In relatively simple scenes, all models detect fruit adequately, but YOLO11-FC2T achieves higher recognition rates, maintaining consistently superior precision across evaluation settings. Unlike YOLOv5 and YOLO10, which occasionally miss low-contrast fruit, YOLO11-FC2T sustains reliable performance. In occluded environments, models such as YOLOv5 and YOLOv8 often miss partially hidden targets or confuse boundaries between adjacent fruit, whereas YOLO11-FC2T delineates cluster contours accurately, markedly reducing missed detections. Under strong illumination and backlighting, YOLOv6 and YOLOv9 tend to misclassify specular reflections as fruit or miss shadowed targets; YOLO11-FC2T suppresses reflection-induced artifacts while preserving subtle edge cues in shadowed regions, demonstrating robust performance against both error modes.

### 5.6 False detection rate

To evaluate the model’s robustness in detecting Taiqiu sweet persimmons during the yellow-green transition period under highly challenging conditions, this study curated a specialized evaluation set of 537 tail-case images. The number of fruits manually marked is 10627. The analysis focused on the comprehensive false detection rate, comprising both false positives (FP: misclassified leaves as fruits) and false negatives (FN: undetected fruits). The false detection rate is calculated as Equation 11:

(11) $$\text{FD Rate}=\frac{\text{FP}+FN}{\text{Total}}\times 100\%$$

This tail-case dataset mirrors high-interference orchard scenarios and is defined by: (1) minimal color separation between foliage and transitioning fruit (HSV difference ≤10); (2) severe illumination variation, including strong specular reflections and backlit shadows; (3) substantial vegetative occlusion (≥40% of fruit area obscured); (4) dense clustering (≥3 fruit per adherent group); (5) small, distant targets (individual fruit area ≤30×30 pixels). Together, these conditions faithfully capture the core difficulties of practical counting in real orchards.

As summarized in **Table 7**, YOLOv5 and YOLOv6 recorded false detection rates of 3.29% and 3.31%, respectively. Structural refinements in YOLOv8 and YOLOv9 lowered these rates to 3.01% and 2.87%, whereas YOLO10, hindered by limited adaptation to small targets, rebounded to 3.14%. Leveraging stronger feature extraction, YOLO11 further reduced the rate to 2.37%. The proposed YOLO11-FC2T achieved the best result at 1.30%, a 1.07 percentage-point reduction relative to YOLO11. Relative to YOLO11, false positives decreased by approximately 49%, effectively curbing overcounting from yellow leaves misclassified as fruit, and false negatives fell by approximately 45%, alleviating missed counts due to occlusion or small, distant targets.

**Table 7:** False detection results on tail dataset.

Model	false positives (FP; leaves misclassified as fruit)	Misses (failure to identify Taiqiu sweet persimmons, FN)	Total (false positives + false negatives)	False detection rate
YOLOv5	108	242	350	3.29%
YOLOv6	78	274	352	3.31%
YOLOv8	63	257	320	3.01%
YOLOv9	71	234	305	2.87%
YOLO10	57	276	333	3.14%
YOLO11	43	209	252	2.37%
YOLO11-FC2T	22	116	138	1.30%

**Figure 11:**
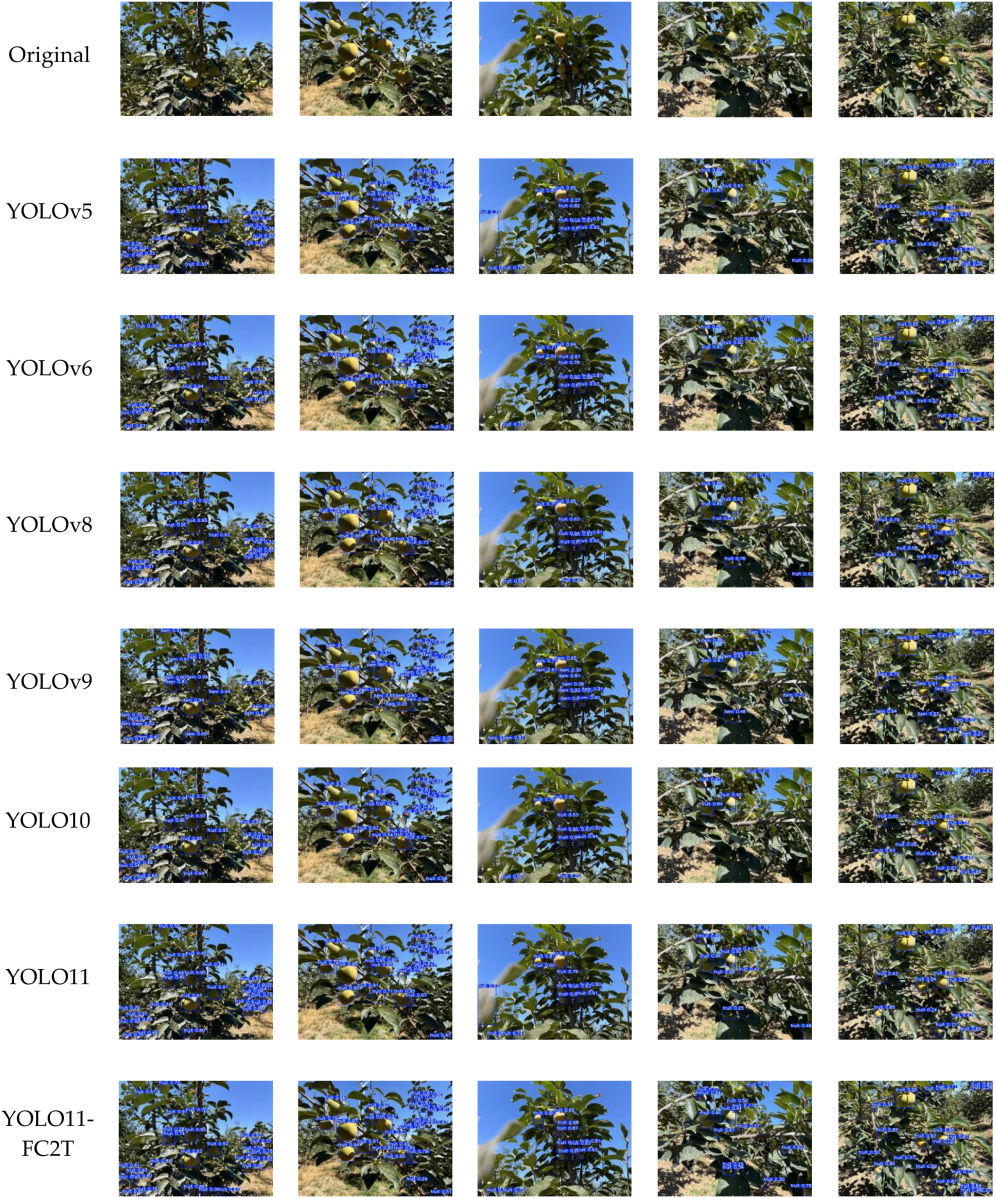
Tail datasets for different models.

To visually substantiate these findings, we randomly selected five images from the tail-case set for qualitative comparison (**Figure 11**), covering five characteristic challenges—strong backlighting with dappled shadows, severe branch–leaf occlusion, dense fruit clusters, distant small fruit, and low fruit–leaf chromatic contrast. Across these scenarios, baseline models consistently produce false positives by misclassifying leaves as fruit and false negatives by missing occluded or small targets. By contrast, YOLO11-FC2T markedly suppresses leaf-induced FP errors while maintaining coverage of small, distant, and partially occluded fruit. This qualitative behavior is consistent with the quantitative results in **Table 7**, reinforcing the enhanced reliability of the proposed model under extremely complex orchard conditions.

### 5.7 Cross-condition test set generalization verification

Although the tail dataset can well cover the high-risk detection scenarios in the orchard during the color change period, this dataset still comes from the same collection area. To further verify the generalization ability of the model in cross-regional and cross-environment conditions, this paper introduces cross-condition test sets for supplementary testing analysis.

To further evaluate the robustness of the model in real application conditions against collection disturbances, this paper introduces a set of cross-condition test sets for supplementary testing. It should be noted that this cross-condition test set is consistent with the self-built dataset of this paper in terms of orchard type, fruit growth stage, and overall scene complexity. The main differences lie in the shooting equipment, shooting location, and specific shooting methods, etc., which lead to subtle deviations in the underlying distribution and imaging characteristics of the images, posing challenges to the model’s generalization ability.

In this experiment, the cross-condition test set did not participate in the model training, validation, or parameter tuning process. This paper directly uses the YOLO11 and YOLO11-FC2T models trained on the self-built dataset for inference testing, without any form of fine-tuning, to ensure that the evaluation results can truly reflect the robustness performance of the model under changes in collection conditions.As shown in **Table 8**, the results of the YOLO11-FC2T model in cross-condition test set inference tests still outperform those of YOLO11. It can be seen that the improved YOLO11-FC2T model has strong generalization ability and is applicable to various situations.

**Table 8:** Cross-condition test set generalization verification result.

Model	P	R	F1	mAP@0.5	mAP@0.5:0.95	False detection rate
Yolo11	90.8%	83.8%	87.2%	93.4%	77.1%	24.6%
Yolo11-FC2T	91.8%	86.8%	89.1%	94.9%	81.1%	12.1%

### 5.8 Heatmap

**Figure 12:**
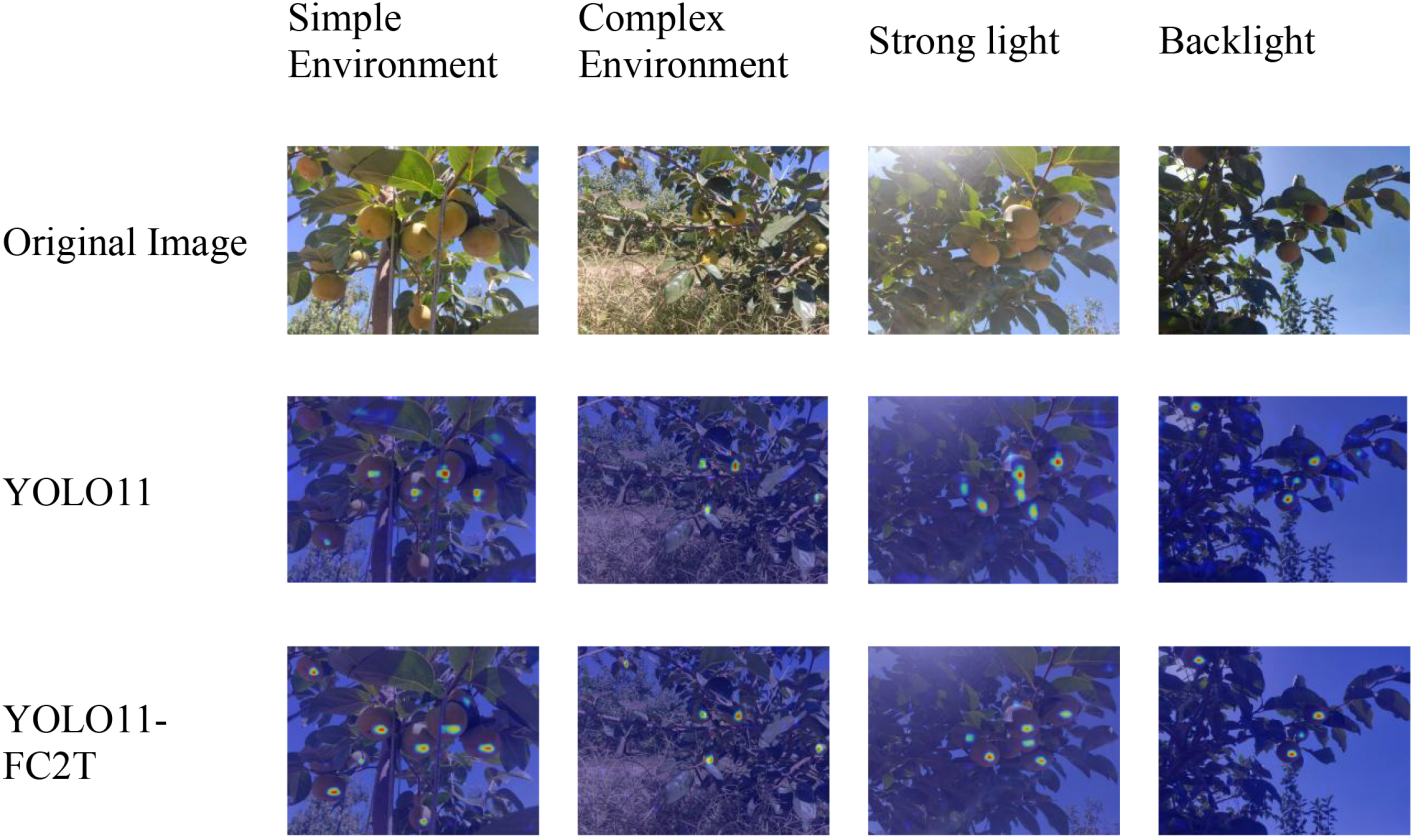
Heatmaps of different models.

This paper generates Grad-CAM heatmaps based on the P3 layer preceding the detection head. It compares the same batch of Taiqiu sweet persimmon samples across four scenarios, as shown in **Figure 12**.

In complex and backlit scenes, YOLO11-FC2T exhibits tighter alignment between its high-response regions and fruit contours/textures than YOLO11, while markedly suppressing activation over leaves and branches. This pattern indicates stronger anti-interference capacity and superior feature localization under the near-color backgrounds and low contrast, thereby reducing leaf–fruit misclassification. Under strong illumination and in uncluttered settings, attention hotspots remain stably concentrated on salient fruit structures rather than specular highlights. In dense or overlapping clusters, activation maps show well-separated multi-peak responses, improving discrimination between adjacent targets and reducing both false-positive activations and missed detections in occluded areas.

### 5.9 Training process visualization

**Figure 13:**
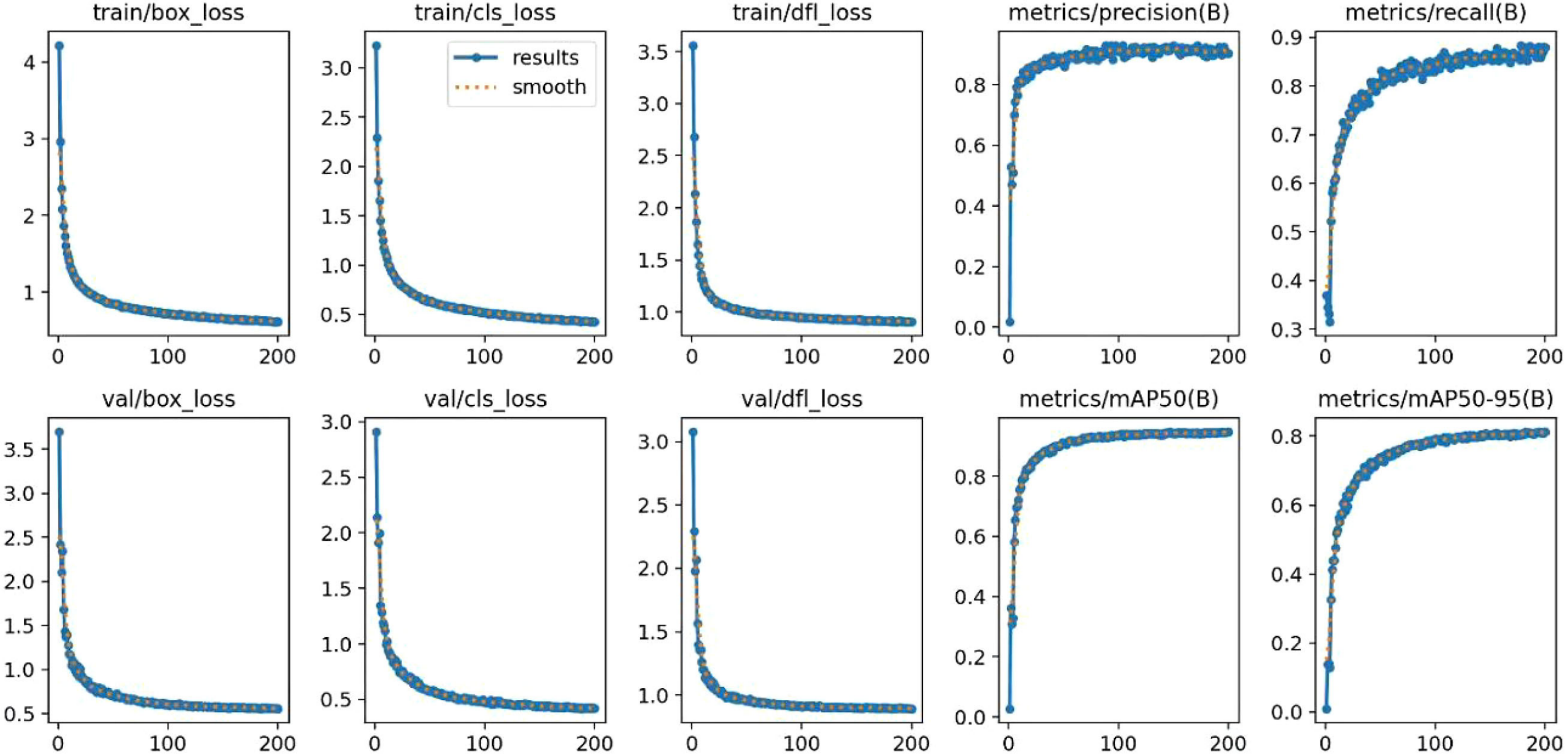
Model training and evaluation results.

**Figure 13** shows the evolution of loss curves and key metrics during training. Both training and validation losses decline steadily with iterations, while precision (P) and recall (R) rise and then stabilize in the final phase, indicating continuous optimization and successful convergence. The training and validation trajectories exhibit closely aligned shapes with minimal separation and no apparent divergence, suggesting limited overfitting and strong generalization. Coupled with the late-stage stability of the performance metrics, these trends confirm that the adopted training configuration guides the model to a favorable solution, delivering reliable performance for practical identification and counting of Taiqiu sweet persimmons during the color-transition.

**Figure 14:**
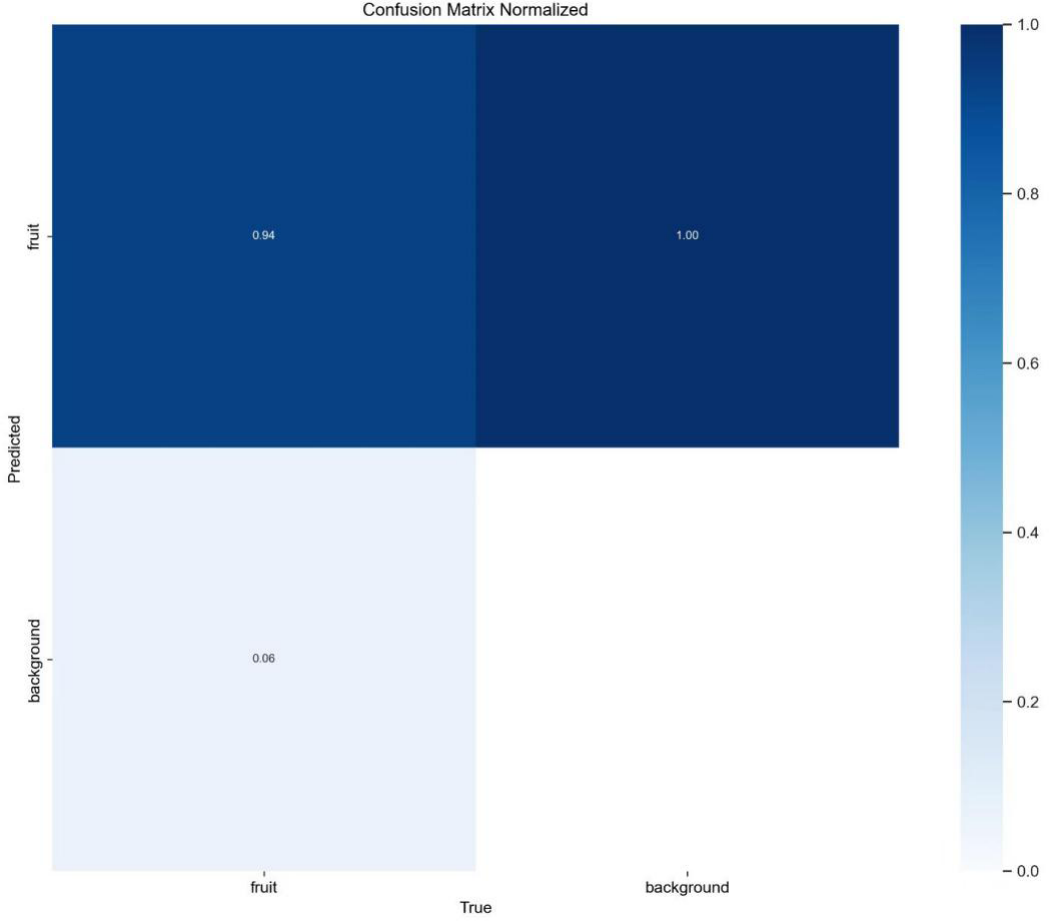
Standardized confusion matrix.

**Figure 14** presents the normalized confusion matrix, showing high recognition accuracy for the Taiqiu sweet persimmon class: dominant diagonal elements indicate that most fruit instances are correctly identified, while off-diagonal entries remain sparse. The model also demonstrates strong background discrimination, with fruit-background confusion occurring primarily under extreme conditions—near-identical hues between yellow leaves and light-colored fruit, strong backlighting, or small, distant targets—manifesting as occasional false positives (leaves misclassified as fruit) or false negatives (missed small fruit). Overall, the confusion matrix corroborates effective recognition during the color-transition while highlighting remaining challenges under severe illumination variation and dense occlusion.

## 6 Conclusion

This study presents an improved YOLO11-based detector tailored to the color-transition of Taiqiu sweet persimmons, delivering a practical, efficient solution for automated fruit identification and counting in this critical phase. The DiffuseMix augmentation strategy generates naturalistic, label-consistent variations that sharpen attention on fruit boundaries and textures while suppressing background activation, substantially reducing both missed detections and false positives under strong illumination and backlighting without additional annotation effort, thereby enhancing generalization and field utility. In concert, four structural enhancements—optimized feature extraction, saliency re-calibration, adaptive multi-scale upsampling, and cross-scale attention fusion—systematically address color-background coupling and the detection of small or adherent fruit, achieving a strong balance between accuracy and computational efficiency suitable for real-time orchard deployment.

Experimental results show that YOLO11-FC2T improves mAP@0.5-0.95 by 4.0% over YOLO11 while markedly enhancing detection accuracy. Although GFLOPs rise slightly and FPS declines moderately, the efficiency trade-offs remain acceptable for practical deployment. Manual verification on a 537-image tail-case set reduced total misdetections from 252 to 138, yielding an MDR of 1.30%. Causal validation via Average Treatment Effect (ATE) indicates that, individually, C3k2_FasterBlock and DySample-T chiefly boost recall, whereas C2PSA_CGA and CAFMAttention primarily elevate mAP@0.5-0.95. Module combinations exhibit clear synergistic gains, and DiffuseMix further strengthens robustness under complex conditions. Collectively, these findings substantiate the complementary benefits of structural optimization and data distribution refinement.

However, the model has not yet been deployed on agricultural machinery for end-to-end field testing. Future work will prioritize in-field deployment during harvest operations, incorporate cross-seasonal data to assess out-of-domain generalization, and refine key architectural components guided by causal diagnostic insights. Despite these constraints, the proposed method provides an effective detection solution for Taiqiu sweet persimmons with strong transfer potential, extendable to maturity assessment and counting of other fruits and vegetables. Overall, it offers both an empirical framework and a methodological reference for intelligent post-harvest management in precision agriculture.

## Data Availability

The original contributions presented in the study are included in the article/supplementary material. Further inquiries can be directed to the corresponding author.
